# Aluminum stress differentially affects physiological performance and metabolic compounds in cultivars of highbush blueberry

**DOI:** 10.1038/s41598-019-47569-8

**Published:** 2019-08-02

**Authors:** María Paz Cárcamo, Marjorie Reyes-Díaz, Zed Rengel, Miren Alberdi, Rebeca Patrícia Omena-Garcia, Adriano Nunes-Nesi, Claudio Inostroza-Blancheteau

**Affiliations:** 1 Programa de Doctorado en Ciencias Agropecuarias, Facultad de Recursos Naturales, Universidad Católica de Temuco, Temuco, Chile; 2 Center of Plant, Soil Interaction and Natural Resources Biotechnology, Scientific and Technological Bioresource Nucleus (BIOREN-UFRO), Universidad de La Frontera, Temuco, Chile; 3 Departamento de Ciencias Químicas y Recursos Naturales, Facultad de Ingeniería y Ciencias, Universidad de La Frontera, Temuco, Chile; 4 Soil Science and Plant Nutrition, UWA School of Agriculture and Environment, The University of Western Australia, Perth, WA Australia; 5 Departamento de Biología Vegetal, Universidade Federal de Viçosa, 36570-900 Viçosa, MG Brazil; 6 Departamento de Ciencias Agropecuarias y Acuícolas, Facultad de Recursos Naturales, Universidad Católica de Temuco, P.O. Box 56-D, Temuco, Chile; 7 Nucleo de Investigación en Producción Alimentaria, Facultad de Recursos Naturales, Universidad Católica de Temuco, P.O. Box 56-D, Temuco, Chile

**Keywords:** Abiotic, Plant physiology

## Abstract

Aluminum (Al) toxicity is one of the major factors that limit the growth and production of crops in acid soils. Highbush blueberry (*Vaccinium corymbosum* L.) cultivars differing in resistance to Al toxicity regarding root growth and photosynthetic performance were used. In this study, we compared the physiological and metabolic strategies to cope with Al toxicity among the highbush blueberry cultivars [two new ones (Camellia and Cargo) and three established ones (Brigitta (Al-resistant), Star and Duke)]. Aluminum concentration in roots and leaves increased in all cultivars after 24 and 48 h of exposure to Al, but less so in roots of cultivar Camellia and leaves of cultivar Cargo. These two cultivars displayed minor effects of Al exposure in terms of photosynthetic activity in comparison with the established cultivars. Furthermore, Cargo did not vary fluorescence parameters, whereas Camellia exhibited a decrease in effective quantum yield (ΦPSII) and electron transport rate (ETR) and a change in non-photochemical quenching (NPQ) and maximum quantum yield (Fv/Fm) under Al after 48 h. The Al treatment increased total phenols in leaves of Brigitta, Cargo, and Camellia, whereas antioxidant activity increased in Star and Cargo after 48 h. Aluminum exposure decreased malate concentration in roots of all cultivars, but no change was noted in fumarate concentration. The antioxidant activity correlated with photosynthetic performance and the total phenol concentration in the leaves of new cultivars exposed to Al, suggesting enhanced resistance in the short-term experiment. The principal component analysis separated the new from the established cultivars. In conclusion, the new cultivars appear to be more Al-resistant than the established ones, with Star being most Al-sensitive. Regarding the Al-resistance mechanisms of the new cultivars, it is suggested that Camellia could have a root Al-exclusion mechanism under Al toxicity. This mechanism could be explained by low Al concentration in roots, suggesting that this cultivar could exude organic acid, allowing to chelate Al in the rhizosphere. Nonetheless, further researches are needed to confirm this assumption.

## Introduction

Acid soils are characterized by nutrient deficiency and toxicity of metals such as manganese (Mn), iron (Fe) and aluminum (Al), with Al toxicity being the main limiting factor for plant growth in acid soils^1^. Aluminum is incorporated into aluminosilicates and other insoluble forms, which are harmless to plants at neutral or near-neutral pH values^2,3^. Aluminum in acidic soils (pH_water_ < 5.0) is solubilized, being available to plants as Al^3+^ and Al(OH)^2+^ forms^4–6^. Acid soils comprise around 50% of the world’s arable lands^7^. Aluminum toxicity to plants includes two categories of responses: (i) short-term responses that can be observed within a few minutes to an hour after Al exposure, and (ii) long-term responses that require hours or days to occur^3,8,9^. However, the Al toxicity effects on plant growth depend on Al concentration, plant species, genotypes, plant age, and growth conditions^1^.

In roots, Al accumulates predominantly in the apical elongation zone, inhibiting cell elongation within a few minutes of Al exposure^10^. The Al-related inhibition of growth and injury to root apex cells has been observed in many plants species^11–13^, including highbush blueberry *Vaccinium corymbosum*^14^. The Al exposure responses are associated with changes in physiological and biochemical processes, including increase in reactive oxygen species (ROS) and damage to biological membranes, as well as negative effects on photosynthetic activity, such as decreases in photosynthetic pigments and fluorescence parameters, reduced enzymatic activity in carbohydrate metabolism, decreased stomatal conductance, and ultimately the programmed cell death^15–18^. In *Citrus*, the CO_2_ assimilation, non-photochemical quenching (NPQ), photochemical quenching (qP), the effective quantum yield of PSII and maximum quantum yield of PSII (Fv/Fm) were decreased by Al toxicity^16^. In *V*. *corymbosum*, a decrease in photosynthetic performance under Al toxicity was noted in the Al-sensitive but not Al-resistant cultivars^19^. Al exposure affected carbohydrate storage, translocation, and metabolism^20^. An increase in carbohydrate concentration in the presence of Al was correlated positively with Al resistance in *Quercus serrata*^21^. In contrast, in highbush blueberry, carbohydrate concentration decreased under Al stress compared with the control^19^. In the Al-sensitive *Citrus grandis*, a decrease in total soluble protein in leaves was reported under Al toxicity, whereas no change occurred in the Al-tolerant species *C*. *sinensis*^22^.

An important mechanism underpinning avoidance of Al stress is the chelation of Al (internally or externally), usually by organic acid anions (OAA) such as citrate, oxalate and/or malate and fumarate (in the order of binding strength OAA:Al)^15,23–26^. In *Populus trichocarpa* and *P*. *tremuloides*, Al-induced exudation of citrate, malate, and oxalate from roots was observed^27^. In addition to OAA, antioxidant compounds such as phenolics also have the capacity to chelate toxic metal ions due to their functional groups [hydroxyl (-OH) and carboxylic (-COOH)]^28^, reducing the harmful effects on plants^29^.

Highbush blueberry grows well in acid soils, with pH_water_ between 4.4 and 5.5^30^. In Chile, this species is usually cultivated in volcanic ash-derived soils^31^, in areas characterized by soil acidity and high availability of Al^3+^, low concentration of exchangeable bases (Ca^2+^, Mg^2+^, K^+^, Na^+^), high rainfall, and severe phytotoxicity of Al^32^. Studies performed in the established highbush blueberry cultivars indicated that short-term Al exposure differentially affects the photochemical features, with Brigitta cultivar showing Al resistance and Bluegold cultivar being Al-sensitive^33^. Besides, in the long-term, cultivar Legacy had higher Al resistance than Bluegold, suggesting different strategies to cope with Al toxicity among these established cultivars^19^. Recently, new cultivars of blueberry such as Camellia and Cargo have been introduced to southern Chile. These cultivars are characterized by early production and high yield during the season, suggesting these new blueberry cultivars are more productive and could be more Al resistant than the established cultivars. Despite the importance of these new highbush blueberry cultivars, there is no knowledge of their Al sensitivity/resistance under acidic conditions and Al toxicity. Thus, this study aimed to compare the physiological and metabolic strategies of coping with Al toxicity between the new and established highbush blueberry cultivars.

## Materials and Methods

### Plant materials and growth conditions

In this study, we used three established cultivars (Brigitta, Star, and Duke), and two new cultivars (recently introduced from USA) (Camellia and Cargo) of highbush blueberry (*Vaccinium corymbosum* L.). One-year-old plants with 40 cm in height were conditioned in plastic pots containing 18 L of Hoagland solution^34^ for two weeks. The composition of this nutrient solution was 3.0 mM KNO_3_, 2.0 mM Ca(NO_3_)_2_, 1.0 mM MgSO_4_, 0.1 mM KH_2_PO_4_, 1.0 mM NH_4_NO_3_, 20 µM Fe-EDTA, 25 µM H_3_BO_3_, 10 µM MnSO_4_, 0.4 µM CuSO_4_, 2.0 µM ZnSO_4_, and 0.07 µM (NH_4_)_6_Mo_7_O_24_; it was renewed every 3 days. The growth chamber conditions were 16/8 h light/dark photoperiod, 22 ± 2 °C temperature, 70% relative air humidity and light intensity around 300 μmol photons m^−2^ s^−1^. The treatments were no Al (control treatment) and 200 µM AlCl_3_ at pH 4.5 adjusted daily; this is a toxic concentration for highbush blueberry as observed in previous studies^19,33^. The physiological parameters were evaluated after 24 and 48 h of Al, the times considered short-term exposure to Al^3+^ for woody plant species^1,20,33^. At these times, fully-expanded leaves and root tissues were harvested for metabolic analyses at the mid-point of the light period. The samples were immediately frozen in liquid nitrogen and stored at −80 °C until further analysis.

### Determination of Al concentration

Aluminum concentration was analyzed as described previously^35^. For this, 1.0 to 3.0 g of dried tissues were ground, dry-ashed in a muffle furnace at 500 °C for 8 h and digested with 2 M HCl. The concentration of Al was determined using a multi-element atomic absorption spectrophotometer (EAA, Model 969, Unicam, Cambridge, UK).

### Gas-exchange and chlorophyll a fluorescence parameters

Photosynthesis-related parameters were determined in fully-expanded leaves as described previously^36^. Shortly, the measurements were performed in the morning using a portable infrared CO_2_ analyzer (Licor LI6400, Lincoln, NE, EUA), equipped with a measurement cuvette with its light source (300 µmol photons m^−2^ s^−1^), and control of temperature (20 °C) and CO_2_ (400 mL/L) according to Reyes-Díaz *et al*.^36^. Chlorophyll *a* fluorescence parameters measured in leaves at the second to fourth shoot node were used to determine the effective quantum yield of PSII using a portable pulse-amplitude-modulated fluorimeter (FMS 2; Hansatech Instruments, King’s Lynn, UK) according to Reyes-Díaz *et al*.^33^. The fluorescence parameters of effective quantum yield (*Ф*_PSII_), electron transport rate (ETR), and non-photochemical quenching (NPQ) were estimated as described previously^37^.

### Determination of photosynthetic pigments

Chlorophyll *a* and *b* and carotenoids were extracted with 100% acetone at 4 °C under safe green light and centrifuged at 10,000 rpm at 4 °C according to Lichtenthaler and Wellburn^38^. Pigments were quantified according to García-Plazaola and Becerril^39^ using phase-reversed solvent-gradient high-performance liquid chromatography (HPLC, Agilent Technologies Inc., San Jose, California, USA).

### Antioxidants assays

The antioxidant activity (AA) in roots and shoots was determined based on the method described previously^40^ using the 2.2-diphenyl-1-picrylhydrazyl (DPPH) free radical scavenging assay. Plant samples were ground in liquid nitrogen and soaked in 1 mL of 80:20 (v/v) methanol:water. The absorbance was measured at 515 nm by a spectrophotometer (UNICOR 2800 UV/VIS, Spain) using Trolox as the standard. The values were expressed in μg Trolox equivalents g^−1^ fresh weight (FW).

### Total phenols

The total phenols (TP) were determined by the Folin-Ciocalteau method, as described by Slinkard and Singlenton^41^. Absorbance was measured at 765 nm and expressed in chlorogenic acid equivalents (CAE) g^−1^ FW.

### Metabolite analyses

Approximately 15 mg of dry ground material was used for metabolite analyses. Samples were subjected to methanol extraction without Ribitol, according to Medeiros *et al*.^42^. The methanol soluble phase was transferred to a 1.5 mL tube for the quantification of sugars, organic acids, and amino acids. The resulting pellet was subjected to three washes with the same extracting solution. Starch and total protein concentrations were quantified in the pellet obtained^43,44^. The supernatants and pellets were stored at −20 °C until further analyses.

The starch and soluble sugars (glucose, fructose, and sucrose) were analyzed as described by Daloso *et al*.^45^ and Stitt *et al*.^46^, with minor modification. The concentrations of total proteins and amino acids were quantified as described by Cross *et al*.^44^. The concentrations of malate and fumarate were determined as described by Nunes-Nesi *et al*.^47^. All measurements were performed in a VersaMaxTM Microplate Reader (Molecular Devices®).

### Experimental design and statistical analyses

The experiment was performed in a split-plot design with five cultivars, three durations of Al exposure, and three replicates. When the data passed the normality and equality of variances after the Kolmogorov-Smirnov test, we performed a two-way analysis of variance (cultivars x duration of Al treatment) and the Tukey test. If data did not pass the Kolmogorov-Smirnov test, the Dunn test and Bonferroni transformations were performed. The Pearson correlation analysis was conducted with a significance level of *P* ≤ 0.05 to examine the relationships among variables. In order to identify the variables that explained the differences between the new and established cultivars, a multivariate analysis by principal components analysis (PCA) was made. All analyses were performed by XLSTAT-base v.2018.5.

## Results

### Aluminum concentration

**Figure 1:**
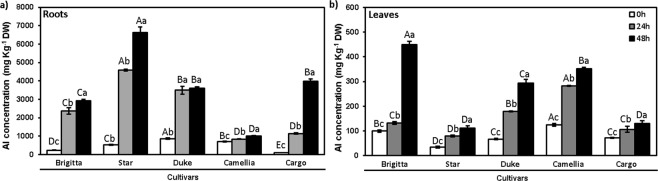
Aluminum concentration in highbush blueberry cultivars under Al toxicity. Aluminum concentration in (**a**) roots and (**b**) leaves after 0, 24 and 48 h of exposure to Al (200 μM Al) in Brigitta, Star, Duke, Camellia, and Cargo cultivars. The values are the average of three measurements per cultivar and treatment. Due to a lack of differences among time points in treatments without Al, we considered the values at 0 h as the average among the start of the experiment and the respective controls for each time point (24 and 48 h). Uppercase letters indicate significant differences (p ≤ 0.05) among cultivars, and lowercase letters indicate significant differences (p ≤ 0.05) among exposure times, according to Tukey test.

The statistically significant interaction between cultivars and duration of Al exposure was noted for Al concentration in roots and leaves (p < 0.001) (Fig. 1). The higher Al concentration was observed after 48 h compared with 24 h in roots and leaves of all cultivars. In new cultivar Cargo, roots exhibited the highest Al concentration at 48 h (39-fold), followed by established cultivars Brigitta (13-fold) and Star (13-fold), whilst in Duke and Camellia an increase in Al concentration was smaller (4- and 1.4-fold, respectively) in relation to their controls (Fig. 1a). In leaves, Brigitta showed the highest Al concentration at 48 h (4.5-fold), followed by Duke (4.4-fold), Star (3.2-fold), Camellia (2.8-fold), and Cargo (1.8-fold) cultivars compared to the respective controls (Fig. 1b).

### Photosynthetic parameters

**Figure 2:**
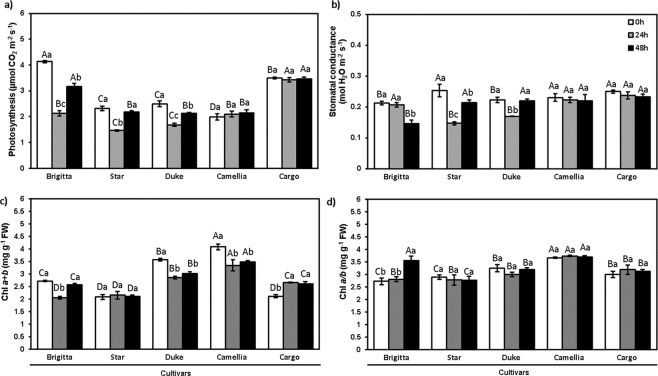
Photosynthesis-related parameters in highbush blueberry cultivars under Al toxicity. (**a**) Photosynthetic rate, (**b**) stomatal conductance (gs), (**c**) Chl *a* + *b*, and (**d**) chlorophylls ratio Chl*a/b* in the control (0 μM Al) and aluminum (200 μM Al) treatments at 24 and 48 h in Brigitta, Star, Duke, Camellia, and Cargo cultivars. The values are the average of three measurements per cultivar and treatment. Due to a lack of differences among time points in treatments without Al, we considered the values at 0 h as the average among the start of the experiment and the respective controls for each time point (24 and 48 h). The bars represent the standard error among replicates. Uppercase letters denote significant differences (p ≤ 0.05) among cultivars, and lowercase letters denote significant differences (p ≤ 0.05) among exposure times, according to the Tukey test.

The significant interaction between cultivars and duration of Al exposure was observed for CO_2_ assimilation (p < 0.001) (Fig. 2a) and stomatal conductance (p ≤ 0.05) (Fig. 2b). The CO_2_ assimilation rate in the established cultivars (Brigitta, Star, and Duke) decreased (by 48, 37 and 32%, respectively) under Al treatment at 24 h, whereas cultivars Star and Duke restored their photosynthesis after 48 h to similar values as the control (Fig. 2a). In new cultivars (Camellia and Cargo), the photosynthesis remained unaltered with respect to the control treatment (Fig. 2a). Stomatal conductance was reduced in Star and Duke (42 and 23%, respectively) after 24 h of Al treatment compared to the control, followed by an enhancement at 48 h (Fig. 2b). In Brigitta, a decrease in stomatal conductance (32%) was noted after 48 h of Al exposure. New cultivars (Camellia and Cargo) did not change stomatal conductance during Al exposure (Fig. 2b).

For all the fluorescence parameters, the significant interaction between cultivars and duration of Al exposure was observed. Concerning the chlorophyll *a* fluorescence parameters, new cultivar Camellia exhibited a significant reduction (p ≤ 0.05) of 40% in *Φ*_PSII_ and ETR at 24 h, whereas established cultivars (Brigitta and Duke) decreased by 32 and 27% after 48 h of Al treatment (Table 1). In Cargo and Star plants, *Φ*_PSII_ and ETR remained unchanged. On the other hand, the NPQ and *F*_v_/*F*_m_ values were unchanged after 24 and 48 h of Al exposure. However, the NPQ values were highest in cultivar Cargo, followed by Star, Camellia, Duke, and Brigitta. *F*_v_/*F*_m_ in all cultivars was around 0.8, which is in the range of healthy values for plants (Table 1).

**Table 1:** Fluorescence parameters in highbush blueberry cultivars under Al toxicity.

Cultivar	ΦPSII			ETR		
	0 h	24 h	48 h	0 h	24 h	48 h
Brigitta	0.20 ± 0.01Ba	0.19 ± 0.02Ba	0.13 ± 0 0.00Bb	24.64 ± 0.72Ba	25.75 ± 0.78Ba	16.64 ± 0.42Bb
Star	0.10 ± 0.00Ca	0.09 ± 0.00Ca	0.10 ± 0.00Ca	12.06 ± 0.43Ca	11.61 ± 0.53Ca	12.10 ± 0.52Ca
Duke	0.21 ± 0.00Ba	0.21 ± 0.00Ba	0.15 ± 0.02Bb	25.92 ± 0.56Ba	25.88 ± 0.13Ba	14.22 ± 0.58Bb
Camellia	0.11 ± 0.00Ca	0.06 ± 0.00Cb	0.09 ± 0.01Ca	13.40 ± 0.21Ca	8.02 ± 0.24Cb	11.01 ± 0.65Ca
Cargo	0.27 ± 0.01Aa	0.27 ± 0.01Aa	0.27 ± 0.01Aa	34.32 ± 0.98Aa	33.87 ± 1.41Aa	33.99 ± 0.66Aa
**Cultivar**	**NPQ**			**Fv/Fm**		
	**0** **h**	**24** **h**	**48** **h**	**0** **h**	**24** **h**	**48** **h**
Brigitta	1.30 ± 0.05 Da	1.26 ± 0.06 Da	1.26 ± 0.04Ca	0.83 ± 0.01Aa	0.82 ± 0.01Aa	0.82 ± 0.00Aa
Star	1.88 ± 0.11Ba	1.98 ± 0.06Ba	2.14 ± 0.09Ba	0.82 ± 0.01Aa	0.82 ± 0.02Aa	0.84 ± 0.01Aa
Duke	1.45 ± 0.06Db	1.40 ± 0.04Db	1.78 ± 0.10Ba	0.81 ± 0.00Aa	0.81 ± 0.00Aa	0.81 ± 0.00Aa
Camellia	1.66 ± 0.03Cb	1.68 ± 0.08Cb	1.95 ± 0.08Ba	0.83 ± 0.01Aa	0.83 ± 0.01Aa	0.84 ± 0.01Aa
Cargo	2.53 ± 0.11Aa	2.50 ± 0.01Aa	2.51 ± 0.16Aa	0.84 ± 0.00Aa	0.84 ± 0.00Aa	0.85 ± 0.01Aa

Effective quantum yield of PSII (ΦPSII), electron transport rate (ETR), non-photochemical quenching (NPQ) and maximum quantum yield of PSII (Fv/Fm) in control (0 μM Al) and aluminum (200 μM Al) treatments. Cultivars tested were Brigitta, Star, Duke, Camellia, and Cargo at 0, 24, and 48 h. The values are the average of three measurements per cultivar and treatment. Due to a lack of differences among time points in treatments without Al, we considered the values at 0 h as the average among the start of the experiment and the respective controls for each time point (24 and 48 h). Uppercase letters indicate significant differences (p ≤ 0.05) among cultivars, and lowercase letters denote significant differences (p ≤ 0.05) among exposure times, according to the Tukey test.

The significant interaction between cultivars and duration of Al exposure was found for chlorophyll pigments. Total chlorophyll content (Chl*a* + *b*) in Brigitta, Duke, and Camellia leaves decreased by 24, 20 and 18%, respectively, after 24 h of Al treatment compared with the control, whereas in Star no significant difference was observed after 24 h of Al exposure. New cultivar Cargo had around a 24% increase in total chlorophyll in the Al treatment (Fig. 2c). In established cultivar Brigitta at 48 h, Chl*a* + *b* recovered to the control values (Fig. 2c). There was no significant difference in the Chl*a*/*b* ratio in all cultivars in the Al treatment, with the exception of an increase in Brigitta after 48 h of Al exposure (Fig. 2d).

**Figure 3:**
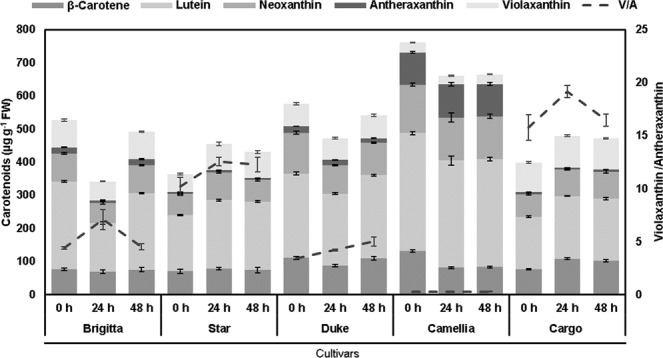
Carotenoid concentrations in leaves of highbush blueberry cultivars under Al toxicity. Carotenoid concentrations and violaxanthin/anteraxanthin ratio in control (0 μM Al) and aluminum (200 μM Al) treatments in cultivars Brigitta, Star, Duke, Camellia, and Cargo at 24 and 48 h. The values are the average of three measurements per cultivar and treatment. Due to a lack of differences among time points in treatments without Al, we considered the values at 0 h as the average among the start of the experiment and the respective controls for each time point (24 and 48 h). Bars represent standard error among replicates. For details and statistical differences, see Supplementary Table 1).

The significant interaction between cultivars and duration of Al exposure was found for leaf carotenoids. New cultivar Camellia had higher concentrations of carotenoids than Duke, Brigitta, Cargo, and Star under Al exposure, whereas Star and Cargo displayed increases. However, cultivars Brigitta, Duke and Camellia exhibited a decrease in carotenoid concentration under Al stress compared to the control (Fig. 3). The leaf *β*-carotene in new cultivar Camellia was decreased (38%) by the Al treatment, whereas in new cultivar Cargo a significant increase (41%) was observed (Fig. 3). Lutein declined significantly (44%) in established cultivar Brigitta at 24 h, increasing afterward. This metabolite increased by around 20% in Cargo and Star under Al toxicity (Fig. 3). Concerning xanthophylls, the new cultivars exhibited unchanged values, whereas the established cultivar Brigitta decreased by about 32% at 24 h, increasing afterward (Fig. 3). In cultivar Star, violaxanthin increased (46%) in the Al treatment, whereas neoxanthin rose by 30% only at 24 h (Fig. 3). New cultivar Cargo increased the violaxanthin/anteraxanthin ratio (V/A) through time, but established cultivar Duke did not vary over time, showing the lower values under Al toxicity than under control. In new cultivar Camellia, the V/A values were lower compared to the other cultivars, whereas in established cultivars Brigitta and Star the V/A values were higher at 24 h compared to the other times (Fig. 3).

### Amino acids and proteins

**Figure 4:**
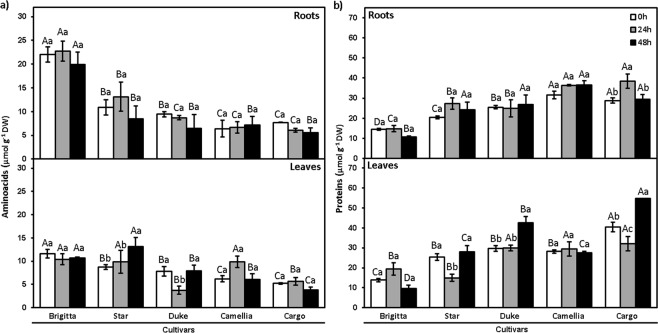
Amino acid and protein concentrations in leaves and roots of highbush blueberry cultivars under Al toxicity. (**a**) Amino acids and (**b**) proteins in control (0 μM Al) and aluminum (200 μM Al) treatments in cultivars Brigitta, Star, Duke, Camellia, and Cargo at 24 and 48 h. The values are the average of three measurements per cultivar and treatment. Due to a lack of differences among time points in treatments without Al, we considered the values at 0 h as the average among the start of the experiment and the respective controls for each time point (24 and 48 h). Bars represent standard error among replicates. Uppercase letters denote significant differences (p ≤ 0.05) among cultivars, and lowercase letters denote significant differences (p ≤ 0.05) among exposure times, according to the Tukey test.

The concentration of amino acids in roots of all cultivars remained constant under Al stress, except in new cultivar Cargo (decreased by 27% at 48 h), with respect to the control (Fig. 4a). In leaves, the interaction between cultivars and duration of Al exposure was significant regarding amino acid concentration. The amino acid concentration in leaves of established cultivar Duke decreased around 52%, and in the cultivar Camellia increased 60%, after 24 h of Al exposure. Amino acids in leaves of established cultivar Star increased 50% after 48 h of Al toxicity (*P* ≤ 0.05), whereas Brigitta did not exhibit significant differences under Al exposure at 24 and 48 h (Fig. 4a).

Protein concentration in roots was similar in all cultivars, with the exception of Cargo at 24 h of Al exposure (Fig. 4b). In leaves, the significant interaction between cultivars and duration of Al exposure was observed for protein concentration. In new cultivar Camellia leaves, protein concentration was unchanged over time, but in established cultivar Star a significant reduction (*P* ≤ 0.05) was noted at 24 h followed by recovery at 48 h. Cargo and Duke had the highest protein concentration at 48 h, whereas Brigitta showed reduced protein concentration after 48 h of Al exposure (Fig. 4b).

### Soluble sugars and starch

Sucrose and starch in roots showed significant interaction (*P* ≤ 0.001) between cultivars and duration of Al exposure, whereas glucose and fructose were significantly affected by the cultivar factor only. Root glucose in the established cultivars did not vary over time, but increased in Camellia by 1.5-fold at 24 h, and diminished by 38% in Cargo at 48 h (Table 2). Regarding root fructose concentration, the most evident change was observed in Camellia (increased 2.4- and 2.2-fold at 24 and 48 h, respectively) (*P* ≤ 0.05) related to the control. In established cultivar Star, an increase in sucrose of 1.5-fold after 24 h and 1.9-fold after 48 h was observed under Al toxicity. In roots, sucrose concentration decreased significantly (by 63 and 93% in new cultivars Camellia and Cargo, respectively) (Table 2). The concentration of starch in Brigitta, Star and Cargo roots was reduced by 33, 71 and 30%, respectively, after 48 h of Al treatment, whereas in Camellia an increase (24%) was noted.

**Table 2:** Soluble sugars and starch in highbush blueberry cultivars under Al toxicity.

Cultivar	Roots			Leaves		
	0 h	24 h	48 h	0 h	24 h	48 h
**Glucose**						
Brigitta	16.50 ± 0.80Aa	15.07 ± 1.81Aa	20.78 ± 4.05Aa	76.33 ± 2.12Ab	73.67 ± 7.25Ab	88.75 ± 1.93Aa
Star	6.53 ± 0.91Ca	10.42 ± 2.72Aa	10.28 ± 1.97Ba	79.96 ± 4.71Aa	69.21 ± 9.36Aa	79.19 ± 8.43Aa
Duke	10.58 ± 0.76Ba	8.63 ± 1.79Ba	8.58 ± 1.24Ba	65.59 ± 3.01Ba	56.55 ± 4.72Ba	64.26 ± 2.59Ba
Camellia	4.72 ± 0.48Cb	8.91 ± 1.28Ba	7.30 ± 1.32Ba	40.19 ± 0.65 Da	36.86 ± 1.78Ca	37.25 ± 1.71 Da
Cargo	15.51 ± 1.06Aa	13.65 ± 0.38Aa	9.66 ± 2.19Bb	52.10 ± 1.06Ca	49.05 ± 4.25Ba	55.39 ± 4.12Ca
**Fructose**						
**Cultivar**	**0** **h**	**24** **h**	**48** **h**	**0** **h**	**24** **h**	**48** **h**
Brigitta	23.62 ± 0.06Aa	22.71 ± 1.99Aa	23.41 ± 2.4Aa	62.67 ± 0.56Aa	62.17 ± 2.12Aa	66.91 ± 2.28Aa
Star	17.34 ± 0.93Ba	20.17 ± 1.83Aa	16.41 ± 1.66Ba	43.19 ± 1.16 Da	47.18 ± 1.04Ba	43.63 ± 1.32 Da
Duke	14.03 ± 0.62Ca	10.82 ± 1.23Cb	10.16 ± 0.63Bb	59.94 ± 0.49Ba	59.99 ± 1.82Aa	59.19 ± 2.14Ba
Camellia	6.03 ± 0.77Db	14.29 ± 0.61Ba	13.44 ± 2.45Ba	52.13 ± 0.67Ca	50.44 ± 1.35Ba	53.93 ± 1.56Ca
Cargo	14.92 ± 0.54Ca	15.03 ± 0.94Ba	13.40 ± 0.83Ba	58.61 ± 0.55Ba	57.55 ± 0.56Aa	59.13 ± 0.27Ba
**Sucrose**						
**Cultivar**	**0** **h**	**24** **h**	**48** **h**	**0** **h**	**24** **h**	**48** **h**
Brigitta	0.18 ± 0.03Ca	0.25 ± 0.13Aa	0.20 ± 0.13Aa	3.04 ± 0.07Aa	2.39 ± 0.34Aa	3.70 ± 0.03Aa
Star	0.17 ± 0.05Ca	0.26 ± 0.06Aa	0.33 ± 0.07Aa	1.16 ± 0.12Ca	1.33 ± 0.48Ba	0.60 ± 0.14Bb
Duke	0.40 ± 0.12Ba	0.46 ± 0.14Aa	0.34 ± 0.11Aa	1.54 ± 0.05Ca	0.59 ± 0.12Cb	0.78 ± 0.52Bb
Camellia	0.54 ± 0.18Ba	0.08 ± 0.08Bb	0.20 ± 0.03Ab	1.14 ± 0.12Ca	1.23 ± 0.10Ba	1.00 ± 0.21Ba
Cargo	1.36 ± 0.18Aa	0.17 ± 0.17Bb	0.09 ± 0.05Bb	2.33 ± 0.28Ba	1.42 ± 0.08Bb	1.17 ± 0.21Bb
**Starch**						
**Cultivar**	**0** **h**	**24** **h**	**48** **h**	**0** **h**	**24** **h**	**48** **h**
Brigitta	10.00 ± 0.25Ba	6.52 ± 0.94Bb	6.88 ± 0.42Bb	33.02 ± 2.79Cb	79.36 ± 12.26Ba	21.49 ± 1.76 Cc
Star	15.20 ± 0.76Aa	12.34 ± 2.72Aa	4.41 ± 2.18Bb	124.38 ± 3.89Aa	118.59 ± 13.58Aa	86.92 ± 13.11Ba
Duke	8.20 ± 0.72Ca	6.00 ± 1.72Ba	7.78 ± 0.61Ba	35.90 ± 5.53Cb	48.37 ± 3.98Cb	66.02 ± 3.35Ba
Camellia	7.32 ± 0.32Cb	9.59 ± 0.82Aa	9.05 ± 0.45Aa	32.33 ± 2.54Ca	11.56 ± 2.20Db	15.35 ± 2.93Cb
Cargo	14.20 ± 0.56Aa	9.98 ± 1.78Ab	9.94 ± 0.48Ab	71.33 ± 4.36Bb	56.74 ± 9.96Cb	113.93 ± 13.57Aa

Glucose, fructose, sucrose, and starch concentration in roots and leaves in control (0 μM Al) and aluminum (200 μM Al) treatments in five cultivars at 0, 24 and 48 h. The values are the average of three measurements per cultivar and treatment. Due to a lack of differences among time points in treatments without Al, we considered the values at 0 h as the average among the start of the experiment and the respective controls for each time point (24 and 48 h). Uppercase letters denote significant differences (p ≤ 0.05) among cultivars, and lowercase letters indicate significant differences (p ≤ 0.05) among exposure times, according to the Tukey test.

In leaves, sucrose (*P* = 0.024) and starch (*P* < 0.001) showed the significant interaction between cultivars and duration of Al exposure. Sucrose decreased in leaves of Star, Duke, and Cargo only. Leaves of Duke and Cargo showed increased, and Brigitta and Camellia decreased, starch concentration under Al exposure (Table 2). In leaves of Star, Duke, Camellia, and Cargo, the concentration of glucose did not change, whereas in Brigitta a slight increase (16%) in glucose was found at 48 h (Table 2). In leaves, fructose was unchanged in all cultivars (Table 2).

### Malate and fumarate concentrations

The significant interaction between cultivars and duration of Al exposure was found for malate concentration in roots (*P* < 0.001). The concentration of malate in roots of all cultivars was reduced by up to 83% compared to the control after 48 h of Al treatment. In leaves, an increase (by 19%) in malate was observed in Brigitta at 24 h (Table 3). Regarding the fumarate concentration, established cultivar Brigitta exhibited changes in roots and especially in leaves, decreasing by 41% in roots at 24 h and by 69% in leaves at 48 h with respect to the control (Table 3).

**Table 3:** Internal organic acid anion concentrations in highbush blueberry cultivars under Al toxicity.

Cultivar	Roots			Leaves		
	0 h	24 h	48 h	0 h	24 h	48 h
**Malate**						
Brigitta	8.61 ± 0.76Aa	4.481 ± 0.86Ab	5.161 ± 1.09Ab	17.00 ± 1.35Ab	21.04 ± 0.47 Aa	19.87 ± 1.66Ab
Star	5.341 ± 0.17Ba	2.211 ± 0.46Bb	1.411 ± 0.14Bc	17.80 ± 1.03Aa	20.70 ± 2.53 Aa	18.44 ± 0.01Aa
Duke	2.371 ± 0.17Ca	1.571 ± 0.33Bb	0.401 ± 0.40Bc	14.16 ± 1.47Aa	14.91 ± 0.35Ba	12.88 ± 0.57Ba
Camellia	1.981 ± 0.09Ca	2.371 ± 0.44Ba	1.311 ± 0.22Bb	14.81 ± 0.96Aa	15.78 ± 1.92Ba	14.68 ± 1.58Ba
Cargo	1.861 ± 0.10Ca	0.981 ± 0.42Bb	0.791 ± 0.45Bb	15.79 ± 1.43Ab	20.74 ± 1.56Aa	16.86 ± 1.56ABb
**Fumarate**						
**Cultivar**	**0** **h**	**24** **h**	**48** **h**	**0** **h**	**24** **h**	**48** **h**
Brigitta	1.231 ± 0.24Aa	0.731 ± 0.11Aa	0.761 ± 0.41Aa	0.51 ± 0.14Aa	0.13 ± 0.08Ab	0.16 ± 0.09Bb
Star	0.551 ± 0.08Ba	0.441 ± 0.22Aa	0.341 ± 0.23Aa	0.20 ± 0.06Ba	0.04 ± 0.26Aa	0.44 ± 0.09Ab
Duke	0.341 ± 0.01Ba	0.491 ± 0.15Aa	0.391 ± 0.18Aa	0.05 ± 0.03Ca	0.00 ± 0.10Aa	0.00 ± 0.00Ba
Camellia	0.561 ± 0.04Ba	0.511 ± 0.12Aa	0.531 ± 0.30Aa	0.00 ± 0.00Ca	0.13 ± 0.01Aa	0.01 ± 0.01Ba
Cargo	0.611 ± 0.03Ba	0.521 ± 0.15Aa	0.711 ± 0.31Aa	0.00 ± 0.00Ca	0.00 ± 0.00Aa	0.00 ± 0.00Ba

Malate and fumarate concentration, and total phenols in roots and leaves in control (0 μM Al) and aluminum (200 μM Al) treatments in five cultivars at 0, 24 and 48 h. The values are the average of three measurements per cultivar and treatment. Due to a lack of differences among time points in treatments without Al, we considered the values at 0 h as the average among the start of the experiment and the respective controls for each time point (24 and 48 h). Uppercase letters indicate significant differences (p ≤ 0.05) among cultivars, and lowercase letters denote significant differences (p ≤ 0.05) among exposure times, according to the Tukey test.

### Antioxidant activity

**Figure 5:**
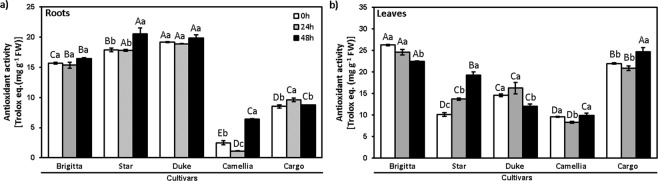
Antioxidant activity in highbush blueberry cultivars under Al toxicity. Antioxidant activity in (**a**) roots and (**b**) leaves in control (0 μM Al) and aluminum (200 μM Al) treatments in cultivars Brigitta, Star, Duke, Camellia, and Cargo at 24 and 48 h. The values are the average of three measurements per cultivar and treatment. Due to a lack of differences among time points in treatments without Al, we considered the values at 0 h as the average among the start of the experiment and the respective controls for each time point (24 and 48 h). Bars represent standard error among replicates. Uppercase letters denote significant differences (p ≤ 0.05) among cultivars, and lowercase letters denote significant differences (p ≤ 0.05) among exposure times, according to Dunn test, Bonferroni correction and Tukey test.

The lowest values of antioxidant activity in roots and leaves subjected to Al toxicity were observed in new cultivar Camellia compared to the other cultivars (Fig. 5). In roots, the interaction between cultivars and duration of Al exposure was significant (*P* < 0.001). The major differences were observed in Camellia roots at 48 h, being 2.5-fold higher than at other time points. Similarly, in the roots of established cultivar Star higher antioxidant activity (1.2-fold) was observed at 48 h compared with other times (Fig. 5a). In leaves of established cultivar Brigitta, there was a significant decrease (14%) at the end of the Al treatment, whereas new cultivar Cargo showed an increase of 12% at the same time (Fig. 5b). Conversely, antioxidant activity in Star increased (35–90%) with Al exposure (Fig. 5b).

### Total phenols

In roots after 48 h of Al exposure, total phenols decreased in Brigitta (12%), Star (66%), Camellia (14%), and Cargo (10%), but not in Duke (Table 4). In contrast, leaves showed an increase in total phenols in Brigitta (67%), Camellia (28%), and Cargo (12%) at 48 h. Conversely, in shoots, significant reductions in this parameter were found in Star and Duke at 24 h under Al stress (Table 4).

**Table 4:** Total phenol concentration in highbush blueberry cultivars under Al toxicity.

Cultivar	Roots			Leaves		
	0 h	24 h	48 h	0 h	24 h	48 h
**Total Phenols**						
Brigitta	0.21 ± 0.01Cb	0.25 ± 0.01Ca	0.18 ± 0.00 Cc	0.75 ± 0.02Db	0.77 ± 0.07Bb	1.26 ± 0.03Ba
Star	0.26 ± 0.01Ba	0.19 ± 0.00Db	0.09 ± 0.01Dc	1.32 ± 0.02Ba	0.89 ± 0.00Bc	1.23 ± 0.01Bb
Duke	0.19 ± 0.00Ca	0.16 ± 0.00 Da	0.18 ± 0.01Ca	1.09 ± 0.04Ca	0.75 ± 0.03Bc	0.98 ± 0.00Cb
Camellia	0.37 ± 0.01Ab	0.64 ± 0.02Aa	0.32 ± 0.00Ab	0.38 ± 0.01Eb	0.34 ± 0.02Cb	0.48 ± 0.03 Da
Cargo	0.29 ± 0.01Bb	0.35 ± 0.03Ba	0.26 ± 0.00Bb	2.19 ± 0.03Ab	2.32 ± 0.05Aa	2.45 ± 0.03Aa

Total phenols in root and leaves in control (0 μM Al) and aluminum (200 μM Al) treatments in five cultivars at 0, 24, and 48 h. The values are the average of three measurements per cultivar and treatment. Due to a lack of differences among time points in treatments without Al, we considered the values at 0 h as the average among the start of the experiment and the respective controls for each time point (24 and 48 h). Uppercase letters denote significant differences (p ≤ 0.05) among cultivars, and lowercase letters indicate significant differences (p ≤ 0.05) among exposure times, according to Dunn test, Bonferroni correction and Tukey test.

### Pearson correlations and principal component analysis

**Figure 6:**
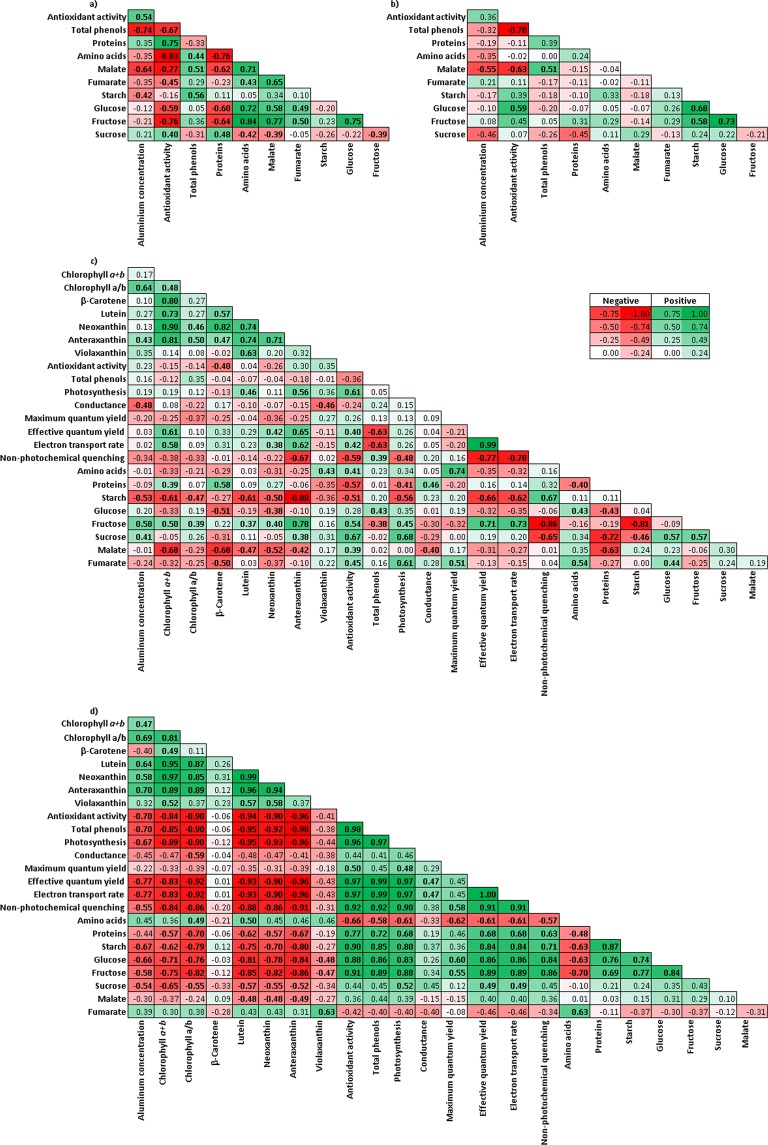
Pearson correlations matrix. Significant correlations coefficients (p ≤ 0.05) are set in bold, with positive and negative correlations being distinguished by green and red, respectively. (**a**) Pearson correlation matrix in roots of established cultivars; (**b**) Pearson correlation matrix in roots of new cultivars; (**c**) Pearson correlation matrix in leaves of established cultivars; and (**d**) Pearson correlation matrix in leaves of new cultivars. Abbreviations: Maximum quantum yield of PSII (Fv/Fm), the effective quantum yield of PSII (ΦPSII), electron transport rate (ETR) and non-photochemical quenching (NPQ).

To evaluate the association between the evaluated features, we calculated Pearson correlation coefficients for all pairs of metabolites at 0, 24, and 48 h of Al treatment (Fig. 6). When the data sets characterizing roots of Brigitta, Star and Duke (established cultivars) were grouped, the most correlations were highly significant compared to new cultivars, Camellia and Cargo (Fig. 6a,b), while this tendency was opposite in leaves (Fig. 6c,d). In roots of all cultivars, the significant negative correlation was observed between Al concentration and malate (Fig. 6a,b). On the other hand, the established cultivars showed a significant negative correlation between Al-concentration and total phenols in roots, whereas a positive correlation was found between Al concentration and antioxidant activity (Fig. 6a). In leaves of the established cultivars, we obtained 14 positive and 21 negative significant correlations, whereas the new cultivars exhibited 54 positive and 57 negative significant correlations. Chl *a* + *b* showed a positive correlation with most carotenoids in all cultivars. In the new cultivars, positive correlations were observed between total proteins and *Φ*_PSII_ or ETR. Hence, these results clearly indicated different physiological and metabolic responses to Al exposure between the established and new highbush blueberry cultivars, as well as different responses in roots and leaves.

**Figure 7:**
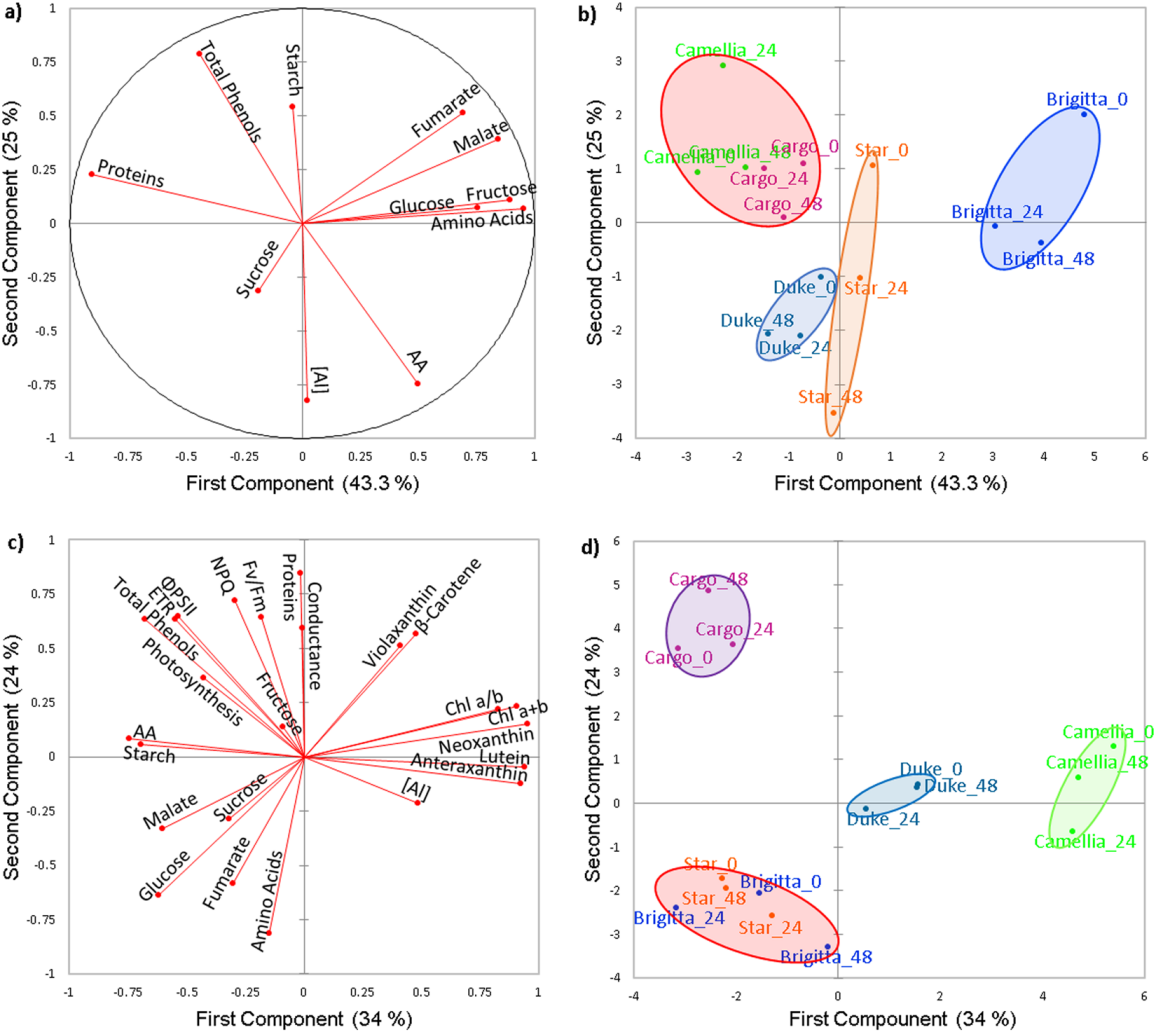
Principal component analysis of physiological and metabolic data of highbush blueberry cultivars. The principal component analysis was performed based on the correlation matrix. Numbers in parentheses give the percent variation explained by the first and the second principal component. Figures (**a**,**c**) show the loading plots, and b and d the score plots obtained from resulting distribution for roots and leaves, respectively. Color circles in the figures (**b**,**d**) represent the clusters formed by Pearson distance.

For PCA, the data obtained for all cultivars were averaged and normalized, as indicated in Fig. 7. For root tissues, the first principal component (PC1), which explained 41.9% of the total variance, included total phenols, starch, and proteins as the main contributing variables (Fig. 7a). The second principal component (PC2) explained 30.6% of the total variance and grouped fructose, glucose, amino acids, malate, and fumarate (Fig. 7a). When we compared the PCA score plots (Fig. 7b) for roots, we observed a clear separation between established (Brigitta, Star, and Duke) and new (Camellia and Cargo) cultivars of highbush blueberry (Fig. 7b), which is very important given that root tissues are the first targets of Al toxicity.

For all studied parameters evaluated in leaves, PC1 and PC2 explained 33.9 and 23.97% of the total variance, respectively (Fig. 7c). The first principal component (PC1) included chlorophyll *b*, chlorophyll *a*, chlorophyll *a* + *b*, neoxanthin, lutein, anteraxanthin, fructose, and Al concentration (Fig. 7c). The PCA score plot (Fig. 7d) for leaves showed a clear separation among the cultivars of highbush blueberry (Fig. 7d).

## Discussion

Impairment in root growth is a primary symptom of Al toxicity and has been used to establish differences in Al sensitivity among cultivars^15,20^. In the roots apexes, Al accumulates in the cell wall due to the trivalent Al cation binding to negative wall charges^48^. Al-tolerant genotypes of wheat accumulated 3- to 8-fold less Al in the root apex than Al-sensitive genotypes^8^. The previous report on highbush blueberry indicated that Al-concentration was twice higher in the Al-sensitive than Al-resistant cultivar in the long-term experiment^19^. In the study presented here, the lowest Al concentration was observed in the roots of Camellia, followed by Duke, Star, Brigitta, and Cargo (Fig. 1a), suggesting that cultivar Camellia could be the most Al-resistant of the cultivars tested.

In *Citrus reshni* subjected to Al stress, a decline was reported in CO_2_ assimilation, non-photochemical quenching (NPQ), the effective quantum yield of PSII (*Ф*_PSII_), and maximum quantum yield of PSII (*F*_v_/*F*_m_)^16,26^. Similarly, Al inhibited ΦPSII and ETR in *Sorghum*^49^. Moreover, Zhang *et al*.^50^ showed a decrease in chlorophyll content and net photosynthesis in *Glycine max* plants under Al treatment. In *Eucalyptus* sp., it was reported that low pH and Al toxicity provoked a gradual decrease in chlorophyll content, photosynthesis, and transpiration^51^. In highbush blueberry, a significant decrease in photosynthetic performance was reported under Al stress^19,33,36^. Our findings showed a similar trend, with established cultivars Brigitta and Duke showing Al-related decreases in *Ф*_PSII_, ETR (Table 1), photosynthesis and chlorophyll concentration (Fig. 2), whereas new cultivar Cargo did not vary these parameters (except chlorophyll concentration, where an increase was found). Conversely, new cultivar Camellia maintained photosynthesis, but showed decreases in chlorophyll concentration, *Ф*_PSII_, and ETR, suggesting that this cultivar may have compensatory mechanisms to cope with Al stress. In addition, we found that a decrease in photosynthesis in established cultivars (Star and Duke) was concomitant with a reduction in stomatal conductance (Fig. 2a,b). Non-photochemical quenching increased significantly in cultivar Duke, whereas in new cultivar Cargo, this parameter did not change, suggesting Cargo showed Al resistance during 48 h (Table 1). Our results showed that *F*_v_/*F*_m_ did not change at 24 and 48 h under Al toxicity in any of the investigated cultivars over the short-term, showing normal values for plants^52^. This is in agreement with the reports on *Quercus glauca* and *Oryza sativa*, where Fv/Fm remained in a healthy range under long- and short-term Al exposure^53,54^.

It has been documented that Al causes harmful effects in the assimilation of nitrogen and impacts nitrogen metabolism as a whole^55,56^. Besides, Al-tolerant plants growing in acid soils prefer NH_4_^+^ to NO_3_^−^ forms, whereas those growing in neutral or calcareous soils are Al-sensitive and prefer NO_3_^−^ to NH_4_^+ ^^57,58^. Similarly, *Vaccinium angustifolium* (lowbush blueberry), adapted to strongly acidic soils, preferred NH_4_^+^ and was strongly inhibited by NO_3_^− ^^59^. Al toxicity in acid soils may inhibit NO_3_^−^ uptake^56^, suggesting detrimental effects on the concentration of amino acids and proteins. In this study, we observed that protein and amino acid concentrations were unaltered in highbush blueberry roots under short-term Al exposure (Fig. 5a,b). In contrast to our findings, Somers *et al*.^60^ found that roots of Al-tolerant wheat showed an increase in total protein content, whereas roots of Al-sensitive cultivar exhibited no changes, suggesting that these findings are dependent on the plant species studied.

Several studies have demonstrated the accumulation of soluble sugars in response to stress, with the type and concentration depending on the plant species and stress treatments^20,61^. There was evidence that Al increased sugar content in woody and cultivated plants^1,62^. In roots, glucose has been reported as a key energy source to promote root growth under Al toxicity^1^. In our case, the roots of cultivars Camellia and Star significantly increased the glucose concentration at 24 and 48 h, whereas Cargo was constant until 24 h, decreasing afterward (Table 2). The increment of glucose in roots of Camellia and Star could be associated with the strategy to cope with Al toxicity. Similar to our results, studies performed on *Quercus serrata* roots indicated greater glucose accumulation under Al exposure^21^.

Organic acid anions and phenolic compounds have been related to mechanisms of Al resistance due to Al chelation to non-toxic forms^2,63^. Malate in roots was positively correlated with Al resistance in several *Eucalyptus* species^64^. In our experiment, malate concentration in roots decreased in all cultivars at 48 h but stayed unchanged in leaves. A potential reason for internal malate decreasing in roots, mainly in Star and Duke, maybe due to exudation. In contrast, Martins *et al*.^26^ reported that *Plantago* species accumulated citrate, oxalate, malate, and fumarate, which are involved in the internal Al detoxification in plant species such as *Melastoma*, buckwheat, *Hydrangea*, and *Camellia sinensis*^65–68^. In our results, fumarate was present in low concentration and did not change in roots and leaves of all cultivars under Al toxicity, which was in agreement with other reports, suggesting low importance, if any, of fumarate in forming metal-ligand complexes^23,27,69^. It appears that Al exposure decreased internal malate concentration in roots of highbush blueberry cultivars, which could be one of the mechanisms related to Al exclusion.

Phenolic compounds were exuded in Al^3+^-treated *Eucalyptus camaldulensis*, *Melaleuca leucadendra*, and *Melaleuca cajuputi*^70^. We observed an increase in total phenols in roots of new cultivar Camellia at 24 h, whereas there was no change in roots of new cultivar Cargo, and there was an increase in leaves of Brigitta, Cargo and Camellia after 48 h of the Al treatment. Ofei-Manu *et al*.^71^ reported that phenolic compounds in the roots of some woody plant species correlated positively with Al tolerance. In the study presented here, total phenols strongly declined in both roots and shoots of established cultivar Star. For the new cultivars, we suggest that phenols could chelate Al in leaves of Cargo and Camellia, contributing to the maintenance of photosynthesis. It was suggested that polyphenols detoxify Al via chelation due to the high Al affinity to phenols^72^. Moreover, phenolic acids have the capacity to reduce oxidative stress, so they are considered antioxidant compounds^28^. Our findings showed that the established cultivars have higher antioxidant activity in roots than the new cultivars; whereas, in leaves, one established (Brigitta) and one new cultivar (Cargo) had higher antioxidant activity than the other cultivars. Antioxidant activity was significantly and positively correlated with photosynthetic performance and total phenols in leaves of the new cultivars, suggesting resistance to Al toxicity in the short-term. In addition, the PCA analysis separated the new cultivars from the established ones. In conclusion, the new cultivars appear to be more Al-resistant than the established ones, with Star being Al-sensitive and Camellia Al-resistant followed by Cargo. Regarding the Al-resistance mechanisms of the new cultivars, it is suggested that Camellia could have a root Al-exclusion mechanism under Al toxicity due to a low Al concentration in roots, suggesting that this cultivar could be exudated organic acid allowing to chelate Al in the rhizosphere. Nonetheless, further experiments are necessary to confirm this assumption.

## Supplementary information

the Supplementary Information
